# Innate Immune Receptors in Human Airway Smooth Muscle Cells: Activation by TLR1/2, TLR3, TLR4, TLR7 and NOD1 Agonists

**DOI:** 10.1371/journal.pone.0068701

**Published:** 2013-07-04

**Authors:** Anne Månsson Kvarnhammar, Lotta Tengroth, Mikael Adner, Lars-Olaf Cardell

**Affiliations:** 1 Division of ENT Diseases, Department of Clinical Sciences, Intervention and Technology, Karolinska Institutet, Stockholm, Sweden; 2 Institute of Environmental Medicine, Karolinska Institutet, Stockholm, Sweden; University of Leuven, Rega Institute, Belgium

## Abstract

**Background:**

Pattern-recognition receptors (PRRs), including Toll-like receptors (TLRs), NOD-like receptors (NLRs) and RIG-I-like receptors (RLRs), recognize microbial components and trigger a host defense response. Respiratory tract infections are common causes of asthma exacerbations, suggesting a role for PRRs in this process. The present study aimed to examine the expression and function of PRRs on human airway smooth muscle cells (HASMCs).

**Methods:**

Expression of TLR, NLR and RLR mRNA and proteins was determined using real-time RT-PCR, flow cytometry and immunocytochemistry. The functional responses to ligand stimulation were investigated in terms of cytokine and chemokine release, cell surface marker expression, proliferation and proteins regulating the contractile state.

**Results:**

HASMCs expressed functional TLR2, TLR3, TLR4, TLR7 and NOD1. Stimulation with the corresponding agonists Pam_3_CSK_4_, poly(I:C), LPS, R-837 and iE-DAP, respectively, induced IL-6, IL-8 and GM-CSF release and up-regulation of ICAM-1 and HLA-DR, while poly(I:C) also affected the release of eotaxin and RANTES. The proliferative response was slightly increased by LPS. Stimulation, most prominently with poly(I:C), down-regulated myosin light chain kinase and cysteinyl leukotriene 1 receptor expression and up-regulated β_2_-adrenoceptor expression. No effects were seen for agonist to TLR2/6, TLR5, TLR8, TLR9, NOD2 or RIG-I/MDA-5.

**Conclusion:**

Activation of TLR2, TLR3, TLR4, TLR7 and NOD1 favors a synthetic phenotype, characterized by an increased ability to release inflammatory mediators, acquire immunomodulatory properties by recruiting and interacting with other cells, and reduce the contractile state. The PRRs might therefore be of therapeutic use in the management of asthma and infection-induced disease exacerbations.

## Introduction

Pattern-recognition receptors (PRRs) recognize and respond to specific microbial components called pathogen-associated molecular patterns (PAMPs) or endogenous molecules produced by injured or dying cells termed danger-associated molecular patterns (DAMPs). Upon recognition of PAMPs or DAMPs, an immune reaction is elicited in order to protect the host from infection [1], [2]. Activation of PRRs has also been implicated in the pathobiology of asthma. Infections of the respiratory tract by bacteria or viruses may act via these receptors to either prevent or exacerbate the clinical presentation of the disease [3], [4], [5], [6].

The PRR family includes the Toll-like receptors (TLRs), nucleotide-binding oligomerization domain (NOD)-like receptors (NLRs), retinoic acid-inducible gene (RIG)-I-like receptors (RLRs). The most well-known members are the TLRs, a family made up by 10 proteins (TLR1-TLR10) in humans [2]. TLR2 acts as a heterodimer in concert with TLR1 or TLR6, and mediates responses to lipoproteins, lipoteichoic acids, peptidoglycan and zymosan. There are indications that TLR10 also has the ability to form heterodimers with TLR1 and TLR2, but the specific ligand has not yet been identified [7]. TLR3 is involved in the recognition of dsRNA from viruses and the synthetic dsRNA analogue poly(I:C) [8]. TLR4 is the main lipopolysaccharide (LPS) receptor [9], TLR5 recognizes bacterial flagellin [10], while TLR7 and TLR8 mediate responses to viral ssRNA and imidazoquinolines such as imiquimod (R-837) and resiquimod (R-848) [11]. TLR9 responds to bacterial and viral DNA containing unmethylated CpG motifs [12].

The NLR family consists of more than 20 receptors divided into four subfamilies based on their N-terminal domain: NLR family, acidic domain containing (NLRA); NLR family, BIR domain containing (NLRB); NLR family, CARD domain containing (NLRC) and NLR family, pyrin domain containing (NLRP) [13], [14], [15]. The best characterized proteins so far are the NLRC members NOD1 and NOD2. NOD1 detects bacterial cell wall peptidoglycan containing γ-D-glutamyl-*meso* diaminopimelic acid (iE-DAP) found primarily in Gram-negative bacteria, while NOD2 recognizes the muramyldipeptide (MDP) MurNAc-L-Ala-D-isoGln that is conserved in peptidoglycans of Gram-positive and Gram-negative bacteria [16], [17], [18]. Moreover, the RLR family comprises three RNA helicases; RIG-I, melanoma differentiation associated gene-5 (MDA-5) and laboratory of genetics and physiology-2 (LGP-2) [2]. RIG-I recognizes ssRNA that has a triphosphate moiety in its 5′-terminus, along with short blunt dsRNA, whereas long dsRNA and long poly(I:C) are detected preferentially by MDA-5 [19], [20]. Poly(I:C)/LyoVec is a mimic of viral RNA that binds to RIG-I/MDA-5. No ligand is yet defined for LGP-2, but it has been described as a regulator of the RIG-I- and MDA-5-mediated immune responses [1], [21].

Human airway smooth muscle cells (HASMCs) are major effector cells in asthma by affecting airway hyperresponsiveness (AHR), remodeling and inflammation [22]. They have previously been shown to express TLRs [23], [24], [25], suggesting that they might have a role in mediating microbe-induced disease exacerbations. Cultured HASMCs are highly plastic, and can switch between a contractile and a synthetic-proliferative phenotype in response to various stimuli. The latter phenotype is mainly unresponsive to contractile agents, has a reduced expression of contractile proteins, proliferates in response to mitogens and produces extracellular matrix (ECM) proteins and cytokines [22], [26]. The aim of the present study was to characterize the expression and explore the function of TLRs, NLRs and RLRs in HASMCs.

## Materials and Methods

### Reagents

The following PRR ligands were used: Pam_3_CSK_4_, FSL-1, poly(I:C), flagellin, R-837, R-848, iE-DAP, MDP and poly(I:C)/LyoVec from Invivogen (San Diego, CA, USA), LPS (*E. coli*) from Sigma-Aldrich (St. Louis, MO, USA) and phosphorothioate-modified CpG oligodeoxynucleotide 2006 (CpG), 5′-tcgtcgttttgtcgttttgtcgtt-3′, from DNA Technology A/S (Aarhus, Denmark) (all with endotoxins levels <0.125 EU/ml). Recombinant human tumor necrosis factor (TNF)-α was obtained from R&D Systems (Minneapolis, MN, USA). The TLR7-specific antagonist IRS661 (5′-tgcttgcaagcttgcaagca-3′) was purchased from DNA Technology A/S.

### Culture of HASMCs

Primary tracheal HASMCs from 4 donors were obtained from Promocell (Heidelberg, Germany) in passage 2. Primary bronchial HASMCs from one donor were obtained from Lonza (Walkersville, MD, USA) in passage 2. All donors were non-asthmatic and healthy subjects. Cells were cultured in smooth muscle cell growth medium (SMCGM) supplemented with 5% FBS, 0.5 ng/ml epidermal growth factor, 2 ng/ml basic fibroblast growth factor and 5 µg/ml insulin (Promocell), 100 U/ml Penicillin, 100 µg/ml Streptomycin (Gibco, Grand Island, NY, USA) and 0.25 µg/ml Fungizone (Gibco) and used up to passage 7. Medium was changed every 48–72 h. At confluence, HASMC cultures exhibited a typical hill-and-valley appearance. Cells were passaged using trypsin (0.04%)/EDTA (0.03%) and plated at a density of 100,000 cells/ml in complete SMCGM. After 24 h of culture, cells were growth-arrested in serum-free medium for 24 h, and then incubated in SMCGM containing 2% FBS based on previous studies showing that the cells are maintained in good condition while preventing proliferation [27]. Cells were then cultured with or without Pam_3_CSK_4_ (1 µg/ml), FSL-1 (100 ng/ml), poly(I:C) (10 µg/ml), LPS (100 ng/ml), flagellin (1 µg/ml), R-837 (5 µg/ml), R-848 (5 µg/ml), CpG (1 µM), iE-DAP (10 µg/ml), MDP (10 µg/ml), poly(I:C)/LyoVec (1 µg/ml) and TNF-α (10 ng/ml) for 24, 48 or 72 h, unless otherwise indicated. The cells were subsequently used for FACS analysis, real-time RT-PCR and proliferation measurements, whereas the culture supernatants were analyzed by ELISA. All cells were maintained at 37°C in a humidified 5% CO_2_ air atmospheres.

For blocking of TLR7 activity, HASMCs were pretreated with IRS661 (0.175 µM) for 1 h followed by addition of R-837 (10 µg/ml) [28]. After an additional 24-h culture period, cell culture supernatants were saved for cytokine analysis.

### ELISA

Cell culture supernatants were assayed for levels of interleukin (IL)-6 (range 3.1–300 pg/ml), IL-8 (31.2–2000 pg/ml), granulocyte macrophage colony-stimulating factor (GM-CSF) (1–64 pg/ml), transforming growth factor (TGF)-β1 (31.2–2000 pg/ml), eotaxin (15.6–1000 pg/ml) and RANTES (31.2–2000 pg/ml) using ELISA plates from R&D Systems. For the TGF-β1 ELISA, samples were activated in 1 N HCl for 10 min followed by neutralization in 1.2 N H_2_SO_4_/0.5 M HEPES prior to analysis, according to the manufacturer’s instructions. An interferon (IFN)-α multisubtype ELISA (12.5–500 pg/ml) from PBL Biomedical Laboratories (Piscataway, NJ, USA) was used to detect all IFN-α subunits except for IFN-αF.

### FACS Analysis

Flow cytometry analyses were performed on a Coulter Epics XL flow cytometer (Beckman Coulter, Marseille, France) together with the analysis software Expo32 ADC (Beckman Coulter). Cultured cells were analyzed for the expression of cell surface markers using the following mouse anti-human mAbs: intracellular adhesion molecule 1 (ICAM-1)-Fluorescein (clone BBIG-I1) from R&D Systems (Minneapolis, MN, USA) and human leukocyte antigen (HLA)-DR-ECD (Immu-35) from Beckman Coulter. For the detection of PRRs, intracellular staining of HASMCs was performed using the IntraPrep™ Permeabilization Reagent Kit (Immunotech, Beckman Coulter), as previously described [29], [30], [31]. The following mouse anti-human mAbs were used: TLR2-PE (TL2.1), TLR3-PE (40C1285) and TLR4-FITC (HTA125) were acquired from AMS Biotechnology (Abingdon, UK). A polyclonal TLR7-FITC (rabbit, IgG) was obtained from Imgenex (San Diego, CA, USA). Unlabeled NOD1 (rabbit, IgG) and FITC-conjugated goat anti-rabbit IgG were from Abcam (Cambridge, UK). Isotype controls relevant for each Ab were used for background staining. Cells were incubated with Abs or appropriate isotype controls for 15 min at RT.

In addition, for studies of the contractile state, unlabeled rabbit anti-human cysteinyl leukotriene 1 receptor (CysLT1R) and β_2_-adrenoceptor (β_2_AR) pAbs (Abcam) were used together with a FITC-conjugated goat anti-rabbit IgG pAb (Abcam). Cells were incubated with the Abs for 30 min in 4°C. All washing and labeling steps were performed in PBS or PBS supplemented with 2% FBS to avoid unspecific binding.

### Cell Proliferation

The cell number and viability of the cells were quantitatively determined using the alamarBlue® Cell Viability Reagent (Invitrogen, Carlsbad, CA, USA) that measures the conversion of resazurin (blue in color) to resorufin (red). The color change is proportional to the number of living cells. Briefly, 1/10 volume alamarBlue reagent was added to 96-well culture plates containing HASMCs stimulated for 24, 48 and 72 h with various PRR ligands and TNF-α. After a 2-h incubation period at 37°C, the absorbance was measured on a spectrophotometer at 570 nm, with 600 nm as a reference wavelength. The proliferation was depicted as percent difference in reduction between treated and control cells according to the following equation:
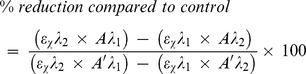



In this formula, ε_ox_λ_1_ and ε_ox_λ_2_ are constants representing the molar extinction coefficients of oxidized alamarBlue at 570 and 600 nm, respectively (ε_ox_λ_1_ = 80586 and ε_ox_λ_2_ = 117216). Aλ_1_ and Aλ_2_ represent the absorbance of the test wells at 570 and 620 nm, respectively, whereas A’λ_1_ and A’λ_2_ correspond to the absorbance of the untreated control wells.

### RNA Extraction and Real-time RT-PCR

HASMCs were lysed in RLT buffer (Qiagen, Hilden, Germany) supplemented with 1% 2-mercaptoethanol. RNA was extracted using an RNeasy Mini Kit (Qiagen), and the quality and quantity of the obtained RNA was determined by spectrophotometry based on the absorbance A_260_/A_280_ ratio. The Omniscript Reverse Transcriptase Kit (Qiagen) and oligo(dT)_16_ primer (DNA Technology A/S, Aarhus, Denmark) were used for cDNA synthesis. The samples were denatured (65°C for 5 min), chilled (4°C for 5 min) and amplified (37°C for 1 h) using a Mastercycler PCR machine (Eppendorf, Hamburg, Germany). Real-time PCR was performed using a Stratagene Mx3000P (Agilent Technologies, Santa Clara, CA, USA). To study PRR expression, FAM™ labeled probes for TLR1-TLR10, NOD1, NOD2, NLRP3, RIG-I, MDA-5, LGP-2 and GAPDH (Applied Biosystems, Foster City, CA, USA) were used as previously described together with Brilliant® QPCR Master Mix (Agilent Technologies) [30], [32], [33]. The thermal cycler was set to perform an initial set-up (95°, 10 min) and 45 cycles of denaturation (95°, 15 sec) followed by annealing/extension (60°, 1 min).

To study the regulation of myosin light-chain kinase (MLCK) upon treatment with PRR ligands, primers for MLCK and GAPDH were designed (Table 1) using Primer Express® 2.0 software (Applied Biosystems) and synthesized by DNA Technology A/S (Aarhus, Denmark). PCR reactions were performed using the Brilliant® II SYBR® Green QPCR Master Mix (Agilent Technologies). The thermal cycler was set to perform 95°C for 15 min, followed by 46 cycles of 94°C for 30 s and 55°C for 60 s (initially 65°C, followed by a 2°C decrease for the six first cycles). Melting curve analysis was performed to ensure specificity of the amplified PCR products. Regardless of method used, the relative amount of mRNA for the genes of interest was determined by subtracting the threshold cycle (Ct) values for the gene from the Ct value for the internal control gene GAPDH (ΔCt). Data are depicted as number of copies of the gene of interest per 100,000 copies of GAPDH (2^-ΔCt^ ×10^5^) [30], [31], [32].

**Table 1:** Sequences of primers used for real-time RT-PCR analysis.

Primer	Sequence
Myosin light chain kinase (MLCK)	Fwd: 5′-GGCCGAGTTGTGGTCAAAGA-3′ Rev: 5′-CAGTGGAACATTTCCCTTGAGC-3′
Glyceraldehyde 3-phosphate dehydrogenase (GAPDH)	Fwd: 5′-AAGCTTGTCATCAATGGAAATCC-3′ Rev: 5′-CAGTGGACTCCACGACGTACTC-3′

### Immunocytochemistry

To determine the presence and subcellular localization of TLR2, TLR3, TLR4, TLR7 and NOD1 in HASMCs, the Dako Cytomation Envision^+^ System horseradish peroxidase (HRP) kit (Copenhagen, Denmark) was used [30]. Briefly, cells were seeded (50,000 cells/chamber) on 4-well Lab Tek™ chamber slides (Nalge Nunc International, Rochester, NY, USA) and grown to 70–80% confluence in complete SMCGM. After two washes in PBS, HASMC were fixed in −20°C methanol or 4% formaldehyde. Slides were then rehydrated in PBS and treated with 0.03% hydrogen peroxide to quench endogenous peroxidase activity. The slides were incubated for 1 h at RT with mouse anti-human mAbs against TLR2 (TL2.3, eBioscience) and TLR3 (40C1285.6, AMS Biotechnology) or rabbit anti-human pAbs against TLR4, TLR7 and NOD1 (Abcam) at a dilution of 1∶10 and 1∶50. Thereafter, they were treated with HRP-labeled goat anti-mouse or goat anti-rabbit polymer for 30 min, followed by 3,3′-diaminobenzidine (DAB) substrate-chromogen for 5 min. Counterstaining was performed with Mayer’s hematoxylin. Thereafter, the glass slides were mounted in Faramount Aqueous Mounting Medium (Dako). As negative controls, N-series universal negative control reagents against mouse and rabbit (both from Dako) were utilized.

### Statistics

Statistical analysis was performed using GraphPad Prism 5 (San Diego, CA, USA). Data are expressed as mean ± SEM, and *n* is equal to the number of independent experiments (passages) performed. For parametric data, statistical differences were determined using paired *t*-test (for two sets of data) or one-way repeated measures ANOVA (for more than two sets of paired data). Non-parametric data were analyzed with Wilcoxon matched-pairs signed rank test. A Grubbs' outlier test was carried out on the replicate analyses to identify possible outliers. *p* values <0.05 were considered statistically significant.

## Results

### Effects of PRR Ligands on the Inflammatory Response

**Figure 1:**
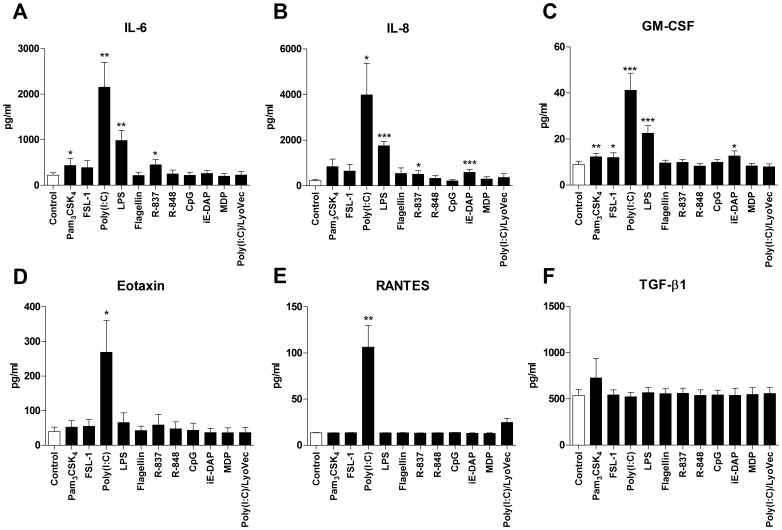
TLR1/2, TLR3, TLR4, TLR7 and NOD1 stimulation promotes cytokine and chemokine release. HASMCs were cultured for 24 h in the absence of presence of Pam_3_CSK_4_ (TLR1/2; 1 µg/ml), FSL-1 (TLR2/6; 100 ng/ml), poly(I:C) (TLR3; 10 µg/ml), LPS (TLR4; 100 ng/ml), flagellin (TLR5; 1 µg/ml), R-837 (TLR7; 5 µg/ml), R-848 (TLR7/8; 5 µg/ml), CpG (TLR9; 1 µM), iE-DAP (NOD1; 10 µg/ml), MDP (NOD2; 10 µg/ml) and poly(I:C)/LyoVec (RIG-I/MDA-5; 1 µg/ml). Thereafter, the cell-free culture supernatants were recovered and analyzed for levels of (**A**) IL-6 (n = 14), (**B**) IL-8 (n = 14), (**C**) GM-CSF (n = 14), (**D**) eotaxin (n = 7), (**E**) RANTES (n = 7), and (**F**) TGF-β1 (n = 6) using ELISA. Data are presented as mean ± SEM. *, p<0.05; **, p<0.01; ***, p<0.001.

**Figure 2:**
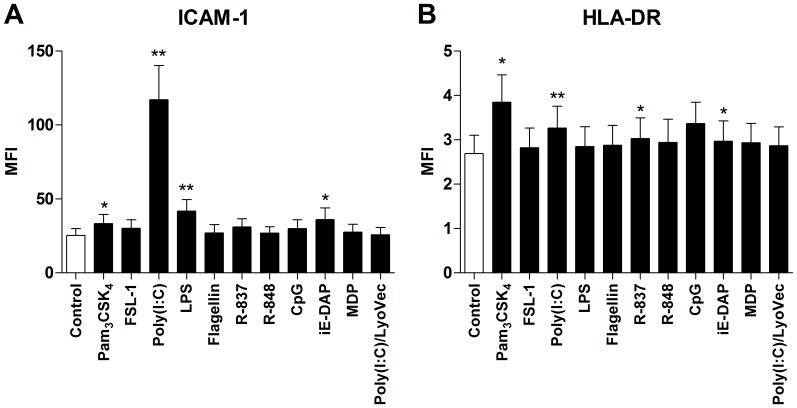
TLR1/2, TLR3, TLR4, TLR7 and NOD1 stimulation induces cell surface marker expression. HASMCs were cultured for 24 h in the absence of presence of Pam_3_CSK_4_ (1 µg/ml), FSL-1 (100 ng/ml), poly(I:C) (10 µg/ml), LPS (100 ng/ml), flagellin (1 µg/ml), R-837 (5 µg/ml), R-848 (5 µg/ml), CpG (1 µM), iE-DAP (10 µg/ml), MDP (10 µg/ml) and poly(I:C)/LyoVec (1 µg/ml). Thereafter, the cells were stained with antibodies against (**A**) ICAM-1 and (**B**) HLA-DR and analyzed by FACS. Data are presented as mean fluorescence intensity (MFI) and presented as mean ± SEM (n = 6). *, p<0.05; **, p<0.01.

In the first set of experiments, the ability of a wide range of PRR ligands to affect cytokine/chemokine release and expression of the cell surface molecules ICAM-1 and HLA-DR was investigated. HASMCs were cultured for 24 h in the absence or presence of Pam_3_CSK_4_ (TLR1/2), FSL-1 (TLR2/6), poly(I:C) (TLR3), LPS (TLR4), flagellin (TLR5), R-837 (TLR7), R-848 (TLR7/8), CpG (TLR9), iE-DAP (NOD1), MDP (NOD2) and poly(I:C)/LyoVec (RIG-I/MDA-5). TNF-α was used as positive control. Poly(I:C) induced a marked release of IL-6, IL-8, GM-CSF, eotaxin and RANTES, but effects were also seen with Pam_3_CSK_4_, FSL-1, LPS, R-837 and iE-DAP on the IL-6, IL-8 and GM-CSF secretion (Fig. 1A-E). The concentration of TGF-β1 was unaffected by PRR stimulation (Fig. 1F) and the level of IFN-α was below the detection limit of the assay both in the absence and presence of the ligands (data not shown). Pam_3_CSK_4_, poly(I:C), LPS, R-837 and iE-DAP also up-regulated the expression of ICAM-1 and HLA-DR (Fig. 2A, B). Remaining ligands affected neither cytokine/chemokine release nor cell surface marker expression. TNF-α induced release of IL-6 (3201±377 *vs.* 219±54.4 pg/ml, p<0.0001), IL-8 (23312±3523 *vs.* 223.6±47.4 pg/ml, p<0.0001), GM-CSF (123.3±40.6 *vs.* 8.9±1.4 pg/ml, p = 0.02), eotaxin (256.3±132.8 *vs.* 39.6±13.0 pg/ml, p = 0.12), RANTES (132.3±20.9 *vs.* 13.6±0.3 pg/ml, p = 0.001) and increased the expression of ICAM-1 (410±48.9 *vs.* 25.3±4.6 MFI, p<0.0001), but not HLA-DR (2.9±0.5 *vs.* 2.7±0.4 MFI, p = 0.33).

**Figure 3:**
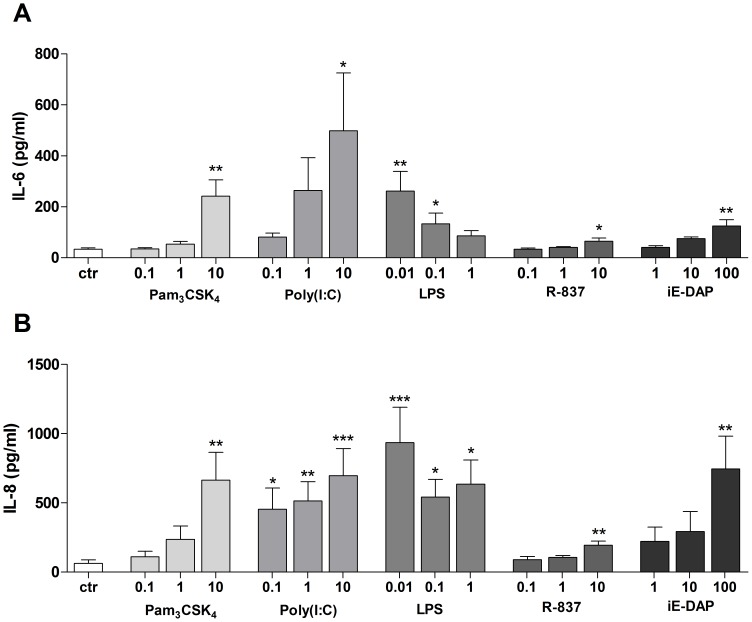
Dose-dependent HASMC activation by Pam_3_CSK_4_, poly(I:C), LPS, R-837 and iE-DAP. Cells were cultured for 24 h with various concentrations of Pam_3_CSK_4_, poly(I:C), LPS, R-837, and iE-DAP followed by measurements of IL-6 (**A**) and IL-8 (**B**) in the cell-free culture supernatants by use of ELISA. All concentration in µg/ml. Data are presented as mean ± SEM (n = 6). *, p<0.05; **, p<0.01; ***, p<0.001.

To further verify that Pam_3_CSK_4_, poly(I:C), LPS, R-837 and iE-DAP induce HASMC activation, cells were cultured with various concentrations of the ligands for 24 h followed by measurements of IL-6 and IL-8 release. A clear concentration-dependent increase was found with Pam_3_CSK_4_, poly(I:C), R-837 and iE-DAP, whereas the opposite was seen with LPS (Fig. 3A-B).

**Figure 4:**
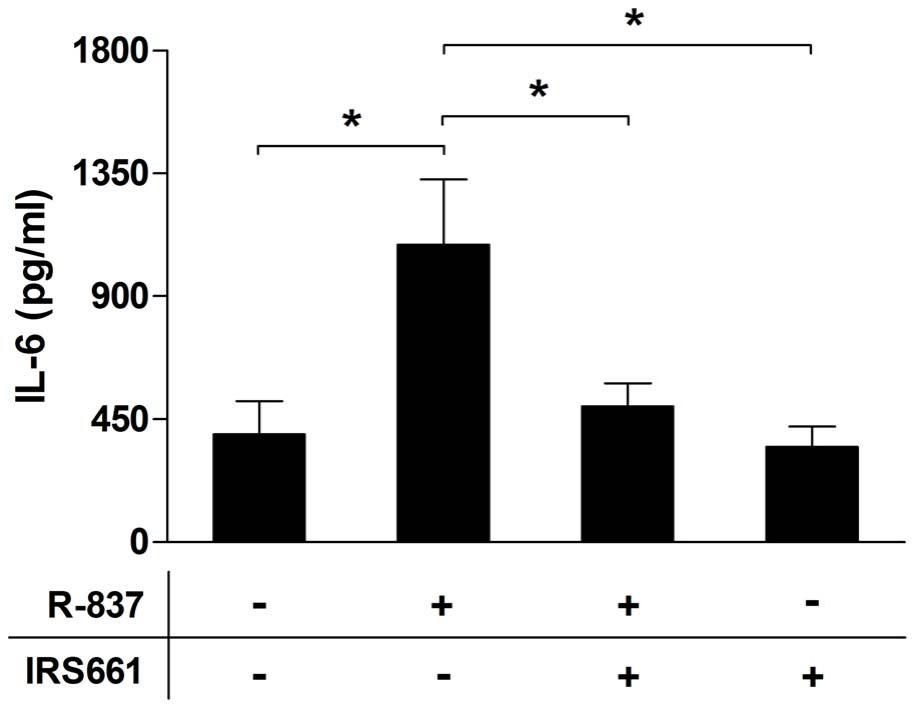
The TLR7 antagonist IRS661 completely abolishes the effect induced by R-837. HASMCs were pretreated for 1 h with IRS661 (0.175 µM) prior to the addition of R-837 (10 µM). After 24 h, the cell-free culture supernatants were recovered and analyzed for levels of IL-6 by ELISA. Data are presented as mean ± SEM (n = 6). *, p<0.05.

Given the low responses to TLR7 stimulation seen and the contradictory findings previously observed in human HASMCs stimulated with TLR7 agonists [23], [24], [34], cells were pretreated with the TLR7 antagonist IRS661 prior to the addition of R-837 to exclude the possibility of off-target effects. After a 24-h incubation period the release of IL-6 was studied in the culture supernatants. Results show that the TLR7 antagonist completely abolishes the R-837-induced activation (Fig. 4).

### Expression of TLR2, TLR3, TLR4, TLR7 and NOD1 in HASMCs

**Figure 5:**
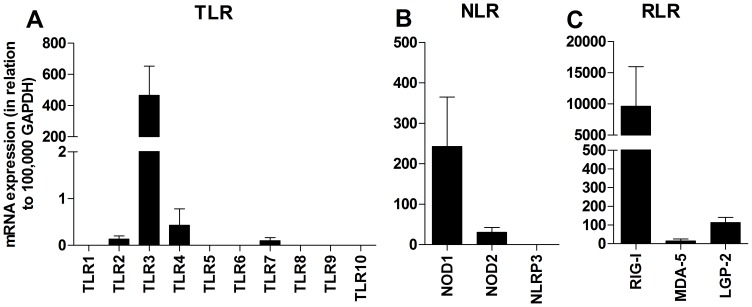
mRNA expression of innate immune receptors in HASMC. The expression of (**A**) TLRs, (**B**) NLRs and (**C**) RLRs was determined by real-time RT-PCR. Data are depicted in relation to GAPDH as 2^-ΔCt^×10^5^ and presented as mean ± SEM (n = 12).

**Figure 6:**
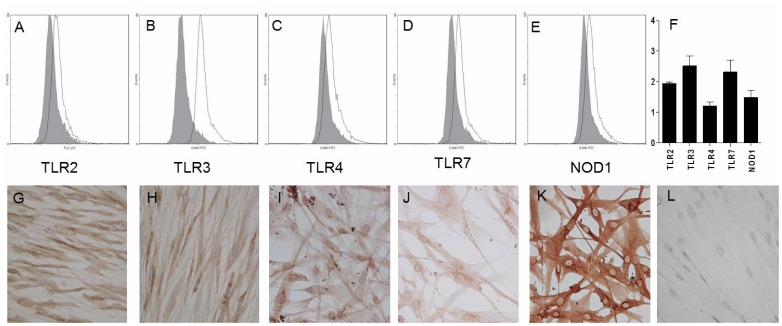
Expression of TLR2, TLR3, TLR4, TLR7 and NOD1 proteins. (**A–E**) HASMCs were stained intracellularly with Abs against TLR2, TLR3, TLR4, TLR7 and NOD1 (open histograms) or appropriate isotype control (shaded histograms) and analyzed by flow cytometry. Data show one out of three independent experiments. (**F**) Data are presented as relative mean fluorescence intensity (rMFI = MFI_Ab_/MFI_isotype control_) and depicted as mean ± SEM (n = 3). (**G**) Immunocytochemistry of HASMCs stained with Abs against TLR2, (**H**) TLR3 (diluted 1∶10), (**I**) TLR4, (**J**) TLR7, (**K**) NOD1 (diluted 1∶50), and (**L**) N-series negative control reagent (diluted 1∶10). Slides were visualized using 3,3′-diaminobenzidine (brown). All slides were counterstained with hematoxylin (blue) and analyzed by microscopy; magnification 200×.

To investigate whether HASMCs express the receptors for Pam_3_CSK_4_, poly(I:C), LPS, R-837 and iE-DAP, real-time RT-PCR was performed using probes for TLR1-TLR10, NOD1, NOD2, NLRP3, RIG-I, MDA-5 and LGP-2. High to moderate levels of TLR3, NOD1, NOD2, RIG-I and LGP-2 was found, along with low levels of TLR2, TLR4, TLR7 and MDA-5. Remaining PRRs could not be detected (Fig. 5A–C). To further verify the functional data, intracellular expression of the receptors was examined by flow cytometry using antibodies against TLR2, TLR3, TLR4, TLR7 and NOD1. HASMCs expressed proteins for all of the receptors investigated (Fig. 6A–F). Moreover, immunocytochemical staining of HASMCs confirmed the presence of TLR2, TLR3, TLR4, TLR7 and NOD1 (Fig. 6G–K). Replacement of the primary specific antibodies with N-series universal negative control reagents resulted in a complete loss of staining (Fig. 6L).

### Effects of PRR Ligands on HASMC Proliferation

**Figure 7:**
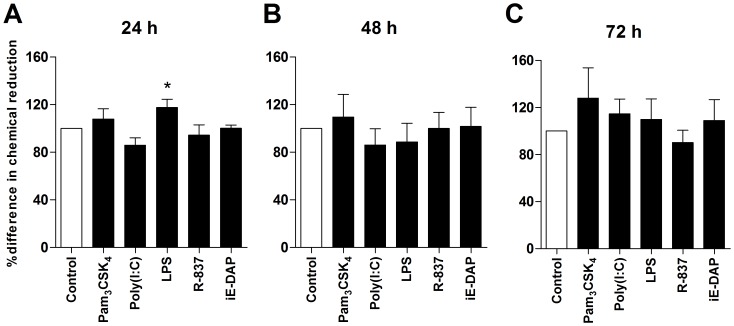
Effects of poly(I:C), LPS, R-837 and iE-DAP on the HASMC viability. (**A–C**) HASMC were cultured for 24, 48 and 72 h in the absence of presence of Pam_3_CSK_4_ (1 µg/ml), poly(I:C) (10 µg/ml), LPS (100 ng/ml), R-837 (5 µg/ml) and iE-DAP (10 µg/ml). Proliferation was quantified with AlamarBlue and determined using a spectrophotometer at 570 and 620 nm. Data are depicted as percent reduction compared to untreated control and presented as mean ± SEM (n = 7–8). *, p<0.05.

The HASMC proliferation upon treatment with suboptimal concentrations of the selected agonists Pam_3_CSK_4_, poly(I:C), LPS, R-837 and iE-DAP was determined using the alamarBlue® assay after 24, 48 and 72 h. A modest increase in proliferation was seen after 24 h upon culture with LPS but not with any of the other ligands (Fig. 7A–C).

### TLR1/2, TLR3, TLR4, TLR7 and NOD1 Stimulation Affects the Contractile Properties of HASMCs

**Figure 8:**
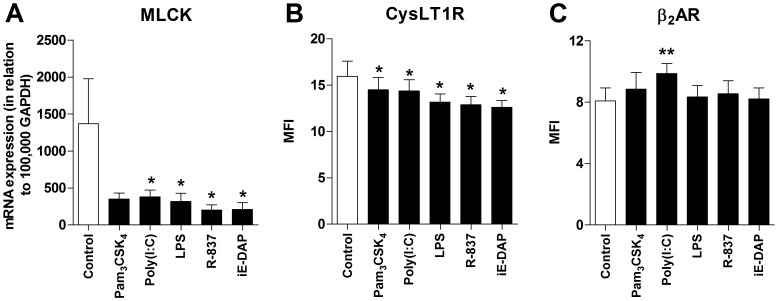
PRR activation affects the contractile state. HASMCs were cultured for 24 h in the absence or presence of Pam_3_CSK_4_ (1 µg/ml), poly(I:C) (10 µg/ml), LPS (100 ng/ml), R-837 (5 µg/ml) and iE-DAP (10 µg/ml). (**A**) The cells were harvested and analyzed for levels of myosin light chain kinase (MLCK) mRNA using real-time RT-PCR. Data are given in relation to GAPDH as 2^−ΔCt^×10^5^. (**B**) Mean fluorescence intensity (MFI) of the cysteinyl leukotriene 1 receptor (CysLT1R) and (**C**) β_2_-adrenergic receptor (β_2_AR) expression using flow cytometry. Data are presented as mean ± SEM (n = 7). *, p<0.05; **, p<0.01.

To explore whether PRR activation affects the contractile state, the expression of myosin, an important protein for the contractile apparatus and a marker for the contractile phenotype [35], and two receptors; the contractile CysLT1R and the relaxant β_2_AR, which both are therapeutic targets in asthma treatment [36], were studied in HASMCs. First, the ability of Pam_3_CSK_4_, poly(I:C), LPS, R-837 and iE-DAP to regulate the mRNA expression of MLCK was assessed by real-time RT-PCR. The expression was significantly down-regulated in HASMCs in response to poly(I:C), LPS, R-837 and iE-DAP (Fig. 8A). Further, the expression of CysLT1R and β_2_AR was studied using flow cytometry. A decrease in CysLT1R expression was seen with all ligands (Fig. 8B), whereas only poly(I:C) up-regulated the expression of β_2_AR (Fig. 8C).

## Discussion

HASMCs are involved in asthma pathogenesis by inducing contraction, proliferation/thickening of the airway wall and generation of lipid mediators, cytokines and chemokines that in turn lead to airway hyperresponsiveness, remodeling and inflammation. Respiratory tract infections are frequent causes of disease exacerbations, and PRRs on HASMCs might have a role in this process. The present study shows that HASMCs express functional TLR2, TLR3, TLR4, TLR7 and NOD1. Stimulation with the corresponding agonists Pam_3_CSK_4_, poly(I:C), LPS, R-837 and iE-DAP, respectively, induces cytokine and chemokine release, up-regulates the expression of cell surface markers and down-regulates the contractile state.

Transcripts for most TLRs have been found in HASMCs, while expression of functional receptors seems to be restricted to TLR2, TLR3 and TLR4 [23], [24], [25], [37], [38], [39], [40]. In rodent models, poly(I:C), LPS, R-837 and R-848 have been shown to alter the contractile ASM responses to a range of contractile agents [5], [6], [41]. No information is currently available regarding NLRs and RLRs in HASMCs. However, vascular SMCs have been reported to express NOD1 and respond to the NOD1 agonist FK565, while being unaffected by NOD2 stimulation [42], [43]. The present study complements and extends previous reports on innate immune receptors in HASMCs by showing mRNA expression for TLR2, TLR3, TLR4, TLR7, NOD1, NOD2, RIG-I, MDA-5 and LGP-2. Remaining TLRs and NLRP3 were completely absent and no effect was seen upon stimulation. However, even though mRNAs for NOD2, RIG-I and MDA-5 were found, the receptors were functionally inactive in these cultured HASMCs. The functionality of LGP-2 remains unknown as there presently is no ligand available. Similar findings when expression and function disconnect have previously been reported for NLRs and RLRs in human T cells and eosinophils [31], [33], and underscores the importance of analyzing receptor functionality in addition to mRNA and/or protein occurrence.

The effects of the PRR agonists were studied on a range of HASMC functions, including release of inflammatory mediators, proliferation and changes in contractility. Initial experiments showed that Pam_3_CSK_4_, poly(I:C), LPS, R-837 and iE-DAP induced secretion of IL-6, IL-8 and GM-CSF, and up-regulated the expression of ICAM-1 and HLA-DR, whereas no/minor effects were seen with FSL-1, flagellin, R-848, CpG, MDP and poly(I:C)/LyoVec. Subsequent experiments revealed that the effects of Pam_3_CSK_4_, poly(I:C), LPS, R-837 and iE-DAP on the proliferative response were small, with only a modest increase with LPS after 24 h. In addition, these agonists down-regulated the contractile state by affecting the expression of MLCK, CysLT1R and β_2_AR. In line with these findings, it has previously been shown that peptidoglycan, lipoteichoic acid (LTA), Pam_3_CSK_4_, dsRNA, poly(I:C) and LPS induce HASMC activation in terms of IL-6, IL-8, macrophage inflammatory protein (MIP)-1α and eotaxin release, and activation of the extracellular signal-regulated kinase (ERK)1/2, p38 mitogen-activated protein kinase (MAPK) and p42/p44 MAPK pathways [23], [24], [25], [38], [39], [40], while no effects are seen with the TLR9 agonist ISS-ODN [38]. Moreover, Lee *et al.* have demonstrated that LTA induces cytosolic phospholipases A2/cyclooxygenase-2-dependent prostaglandin E_2_ and IL-6 generation [44], and promotes the formation of reactive oxygen species and expression of heme oxygenase by human tracheal SMCs [45].

None of the ligands used were found to enhance the production of TGF-β1 or IFN-α. The former plays an important role in tissue repair and in the fibrotic changes occurring within the asthmatic airway [46], [47], suggesting that PRR activation does not contribute to airway remodelling. IFN-α, on the other hand, is a type I IFN usually produced upon triggering of IFN-regulatory factors in response to viral activation through TLR3 and TLR7/8 [2], [48]. The lack of IFN-α release upon stimulation with poly(I:C) and R-837 is somewhat surprising, especially given the strong activation of HASMCs induced via TLR3. Instead, our findings suggest the sole involvement of NF-κB, leading to the generation of a robust pro-inflammatory cytokine response [49].

TLR3 was highly expressed in our HASMC cultures and the most vigorous responses were seen upon stimulation with poly(I:C). In addition to the ability of the TLR3 agonist to induce pro-inflammatory cytokines, it also promoted the release of eotaxin and RANTES. Similar inductions of eosinophilic chemokines by HASMCs in response to TLR3 stimulation have been reported previously [23], [37], [40]. In addition, Niimi *et al.* have shown that culture supernatants of poly(I:C)-stimulated bronchial SMCs increase the chemotactic activity of isolated human eosinophils [37]. Stimulation of TLR3 has also been found to promote CXCL10 generation, production of ECM proteins and expression of ICAM-1 [24], [34].

The present study demonstrates mRNA and protein expression of TLR7 in HASMCs and that R-837 induces activation. Also, the TLR7 antagonist IRS661 completely abolished the cytokine release induced by R-837, suggesting that the activation truly is mediated by TLR7 and not due to off-target effects. Expression of functional TLR7 has been shown in guinea pig ASMCs [5], [6], while results regarding TLR7 in the human setting are equivocal. In line with our data, Kuo *et al.* have reported that R-837 induces HASMC production of the ECM proteins fibronectin and perlecan [34]. In contrast, other groups have shown that the TLR7 mRNA expression is low or even absent in HASMCs under basal conditions and that the cells do not respond to TLR7 agonists. However, in the presence of pro-inflammatory cytokines or in co-culture with peripheral blood mononuclear cells (PBMC), they have been able to induce TLR7 expression and TLR7-mediated cytokine production [23], [24].

PRR activation of HASMC *in vitro*, particularly via TLR3, TLR4 and TLR7, down-regulated the contractile state by affecting the expression of MLCK, CysLT1R and β_2_AR. The function of MLCK is to phosphorylate the myosin light chain, leading to contraction. Elevated levels have been found after sensitization of human airways and in asthmatic compared to normal ASM [22]. The down-regulated mRNA levels seen upon stimulation with poly(I:C), LPS, R-837 and iE-DAP suggest that these agonists have the ability to reduce the MLCK activity, leading to a more non-contractile phenotype [35].

Bronchoconstriction can also be induced by mediators released from inflammatory cells and nerves, such as acetylcholine, histamine and cysteinyl leukotrienes. The latter binds the G-protein coupled receptor CysLT1R to mediate HASMC contraction. Unlike our data showing a decreased expression of CysLT1R in response to Pam_3_CSK_4_, poly(I:C), LPS, R-837 and iE-DAP, Morishima *et al.* have recently shown that poly(I:C) decreased the muscarinic acetylcholine receptor M2R and increased M3R expression without affecting histamine receptor H1R or CysLT1R, which correlated to intracellular calcium mobilization [50]. Possible explanations behind these discrepancies in results can be due to donor differences, different origins of the ASMCs used (tracheal *vs.* bronchial) or methodological variations. Further, poly(I:C) was found to induce β_2_AR expression in our HASMC cultures. β_2_AR binds β_2_-agonists and mediate ASM relaxation via the release of cAMP. The β_2_AR-cAMP axis is abnormally regulated in asthma and is the most well used target of asthma treatment [51], [52]. The up-regulated expression of β_2_AR after TLR3 stimulation further strengthens our finding of down-regulated contractile state by showing that poly(I:C), via separate pathways, both limits contractile receptors and promotes relaxant receptors. However, Cooper *et al.* have shown that poly(I:C) does not affect ASM contractility or relaxation in human small airways [40]. In contrast, other groups have demonstrated that poly(I:C) and LPS enhance the bradykinin-induced contraction of murine or canine tracheal SMCs [3], [53], and that LPS promotes pro-asthmatic changes in the contraction and relaxation responsiveness of isolated rabbit ASM [38]. Moreover, stimulation with R-837 and R-848 has been shown to mediate both acute and long-term effects in reversing ASM contractility induced by electrical field stimulation, 5-hydroxytryptamine, acetylcholine, methacholine, histamine and potassium chloride in guinea pigs [5], [6].

Except for poly(I:C), the responses to some of the ligands were rather weak, which raises questions as to the biological versus the statistical significance. However, culturing isolated HASMCs with specific PRR ligands creates a mono-culture and an isolated system that does not mirror the natural biological environment in which different cell types and mediators interact and cooperate. Efficient detection and clearance of infections is enabled by the fact that several signals and events are triggered simultaneously. Indeed, pro-inflammatory cytokines, including IL-1β, TNF-α and IFN-γ [23], [24], [25], [40], and the Th2 cytokine IL-4 [37] have been found to synergistically enhance the TLR expression levels and/or the TLR3-induced cytokine and chemokine release. In addition, co-culture of HASMCs with PBMC has resulted in a marked cooperative response to TLR2, TLR4 and TLR7/8 stimulation. Moreover, dual stimulation of HASMCs with LPS and poly(I:C) synergistically increased the cytokine generation in the presence of leukocytes [24], [25]. Hence, it is not the effects of a ligand alone, but the combined actions of different cells and signals that are of biological importance. In addition, the fact that the TLR7 antagonist IRS661 abolished the effects of R-837 strengthens the biological relevance of our findings and minimizes the risk of off-target effects and the possible contribution of contaminants.

Activation of HASMCs via TLR2, TLR3, TLR4, TLR7 and NOD1 promotes the development of a synthetic phenotype, characterized by an increased ability of the cells to release inflammatory mediators, acquire immunomodulatory properties by recruiting and interacting with other cells, and reduce the contractile state. It is tempting to speculate, based on our findings together with previous *in vivo* data [54], [55], that a careful manipulation of the PRR system could be of therapeutic use in the management of asthma and infection-induced disease exacerbations.
